# Insomnia in Patients Seeking Care at an Orofacial Pain Unit

**DOI:** 10.3389/fneur.2019.00542

**Published:** 2019-05-28

**Authors:** Miguel Meira e Cruz, Nenad Lukic, Aleksandra Wojczynska, Beat Steiger, Antonio Sérgio Guimarães, Dominik A. Ettlin

**Affiliations:** ^1^Sleep Unit, Cardiovascular Center, School of Medicine, University of Lisbon, Lisbon, Portugal; ^2^Interdisciplinary Orofacial Pain Unit, Faculty of Medicine, Center of Dental Medicine, University of Zurich, Zurich, Switzerland; ^3^Laboratório Experimental de Dor, Faculdade de Medicina e Odontologia, São Leopoldo Mandic, São Paulo, Brazil

**Keywords:** orofacial pain, sleep, insomnia, dysfunction, prevalence, insomnia severity index, sleep questionaire, epidemiology

## Abstract

**Introduction:** Orofacial pain and dysfunction include a broad range of disturbances among which pain and insomnia are some of the most common complaints. Sleep strengthens physiological and psychological resilience and is an absolute requirement for health. Insomnia is a common symptom or sleep disorder, yet data on its prevalence is sparse. Here we extracted data from the insomnia severity index which was part of the web-based interdisciplinary symptom evaluation (WISE) tool given to a large sample of patients seeking care at an orofacial pain unit for analyzing insomnia prevalence in this clinical population.

**Material and methods:** Anonymized data were available from 952 patients who consulted the Orofacial Pain Unit at the Center of Dental Medicine, University of Zurich, Zurich, Switzerland between January 2017 and December 2018. Prevalence data for insomnia stratified by gender and 10 age groups (decades) were calculated. The distribution of four insomnia severity grades was determined, also stratified by age and gender.

**Results:** 952 patients (290 men: 30.5%) with a mean age of 44.8 ± 17.4 years completed a WISE. Three hundred and fifty-two (37.0%) patients with a mean age of 45.8 ± 16.7 years positively responded to a screening question for insomnia and/or hypersomnia. Insomnia was severe in women from the 2nd to 8th decade, ranging from 4.3% (3rd decade) to 14.5% (6th decade), and moderately severe from the 2nd to 9th decade, ranging from 18.8% (6th decade) to 27.8% (2nd decade). In men, severe insomnia was present from the 3rd to 7th decade, ranging from 2.3% (7th decade) to 4.4% (4th decade) and moderately severe insomnia from the 3rd to 7th decade, ranging from 4.6% (7th decade) to 12.2% (5th decade).

**Conclusions:** This is the first study reporting on insomnia in a large sample of patients seeking care at an orofacial pain unit. One in three patients reported some form of sleep disturbances, which for almost half of them was moderate to severe insomnia. The gender ratio was almost equal throughout adulthood, yet younger and older women were more frequently affected and experienced higher insomnia severity than men.

## Introduction

Orofacial disorders can be painful or painless and with or without functional impairment. They include a broad range of disturbances with heterogeneous etiologies in dental, mucosal, musculoskeletal, and neuronal tissues. These conditions can be summarized under the umbrella term orofacial pain and dysfunction (OPD). According to community based surveys, the prevalence of OPD varies greatly, from 5 to 57% depending on the study period, population, location, and other factors (1). Chronic OPD affect women more frequently (2, 3). Impaired sleep and insomnia are commonly reported by patients with OPD (4, 5).

Healthy sleep was recently defined in a joint consensus statement of the American Academy of Sleep Medicine and Sleep Research Society as “adequate duration, good quality, appropriate timing and regularity, and the absence of sleep disturbances or disorders” (6). Sleep strengthens physiological and psychological resilience (7). Sleep regulation has a sleep-promoting, homeostatic component and a circadian component (8, 9). When a person sleeps or is awake (circadian sleep propensity) characterizes the individual chronotype. This is in part determined by genetics, and modified by age, activity, and light exposure (10–12). In the long-term, disturbed sleep is associated with multiple adverse health outcomes, including cardiovascular, metabolic, and mental health disturbances (13–16). The most common sleep disorder is insomnia, defined as “a persistent difficulty with sleep initiation, duration, consolidation, or quality that occurs despite adequate opportunity and circumstances for sleep” (17). Hence insomnia is vague term describing the phenotypes of several sleep disturbances with different underlying etiologies. Different studies of non-clinical populations worldwide assessments have reported an insomnia prevalence from 5 to 50%, mostly with a predominance in females (18–25). For insomnia evaluation, subjective assessment is considered the gold standard and has been recommended for the evaluation of patients with OPD (26–29). Numerous relatively brief self-reported questionnaires have been developed and validated to detect and quantify insomnia or sleep-related impairment in different populations (30, 31). The Patient Health Questionnaire 9 (PHQ-9) commonly used in primary care to screen for depression includes the item “trouble falling or staying asleep or sleeping too much” that screens for insomnia and/or excessive daytime sleepiness (32). In addition to screening, the insomnia severity index (ISI) also offers some quantification. (33, 34). This brief self-report questionnaire consists of seven items to be rated on a five-point Likert scale, ranging from 0 (none) to 4 (very severe). The items are difficult initiating and maintaining sleep; awaking early; dissatisfaction with current sleep patterns; sleep related impairment of quality of life (noticed by others), worries or distress, and interference with daytime functioning. Total scores range from 0 to 28, whereas higher scores indicate more severe insomnia. Four insomnia grades have been categorized (scores in brackets): not clinically significant (0–7), subthreshold (8–14), moderate (15–21), and severe (22–28). Moderate to severe grades are considered clinically relevant.

For this paper, we used data collected by the web-based interdisciplinary symptom evaluation (WISE) which is a new self-report instrument designed to obtain computer guided patient information related to orofacial disorders of diverse etiologies and comorbid conditions (35). The WISE combines a symptom-burden checklist with various validated psychometric in-depth questionnaires serving as case-finding instruments, one of them being the ISI. Insomnia associated with chronic pain is phenotypically similar to primary insomnia (36). Yet the interaction of sleep and pain is poorly understood (37–39). High quality prevalence data of insomnia in persons suffering from pain (including OPD) is sparse (40, 41). In this work, we aimed at identifying the prevalence of insomnia in a large sample of patients seeking care at an orofacial pain unit.

## Material and methods

Anonymized data were extracted from WISE completed by 952 patients who consulted the Orofacial Pain Unit at the Center of Dental Medicine, University of Zurich, Zurich, Switzerland between January 2017 and December 2018. Patients completed the WISE prior to their first appointment. The symptoms of our study population varied from painless yet burdening functional disorders to painful conditions with widely varying orofacial pain location, quality, time pattern, and related disability.

For questionnaires to be analyzed, patients must have clicked a checkbox indicating their consent that their anonymized data can be used for research. According to Swiss law, the analysis of strictly anonymized data does not require approval by an ethics committee. We compared the clinic population that completed the WISE with the clinic population that did not complete it or did not give consent to use the data for research.

Using the software SPSS version 23, we calculated prevalence data for insomnia stratified by gender and age group (decades). The distribution of four insomnia severity grades was determined stratified by age and gender. Means were compared between genders using an independent sample *t*-test with Welch's correction, since equality of variance was not assumed.

## Results

**Figure 1:**
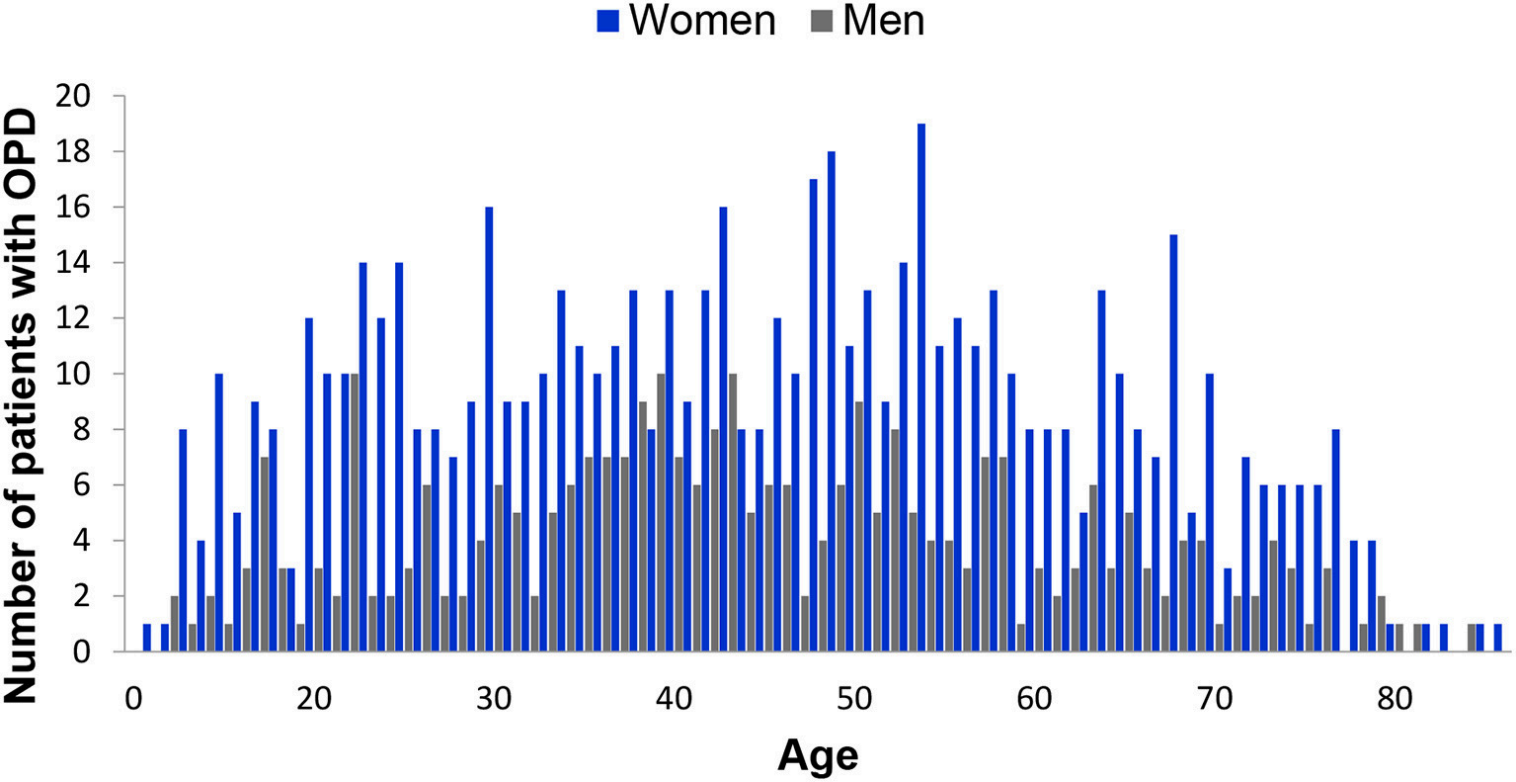
Age and gender distribution of the global sample of 952 patients experiencing orofacial pain and dysfunction (OPD).

**Figure 2:**
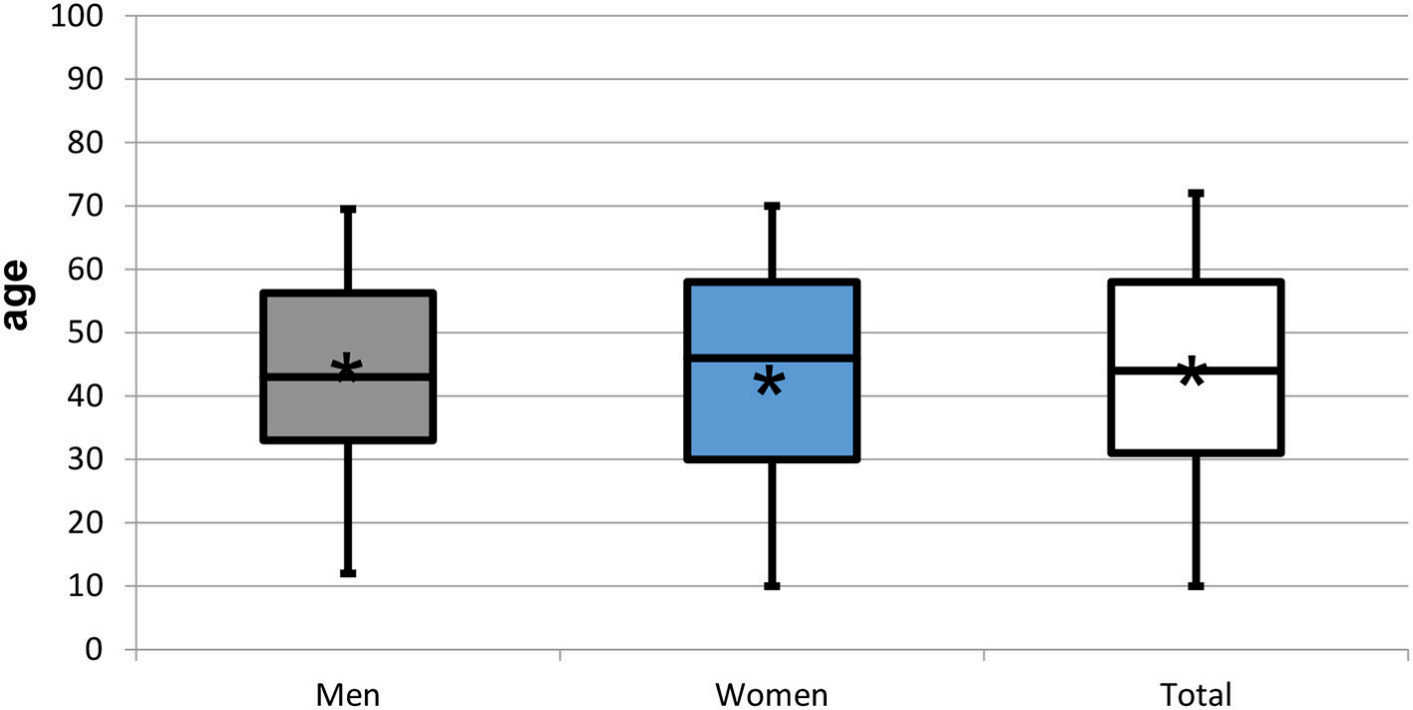
Box plots representing the age distribution by gender of the 952 patients experiencing orofacial pain and dysfunction. Star symbol (^*^) indicates mean.

**Figure 3:**
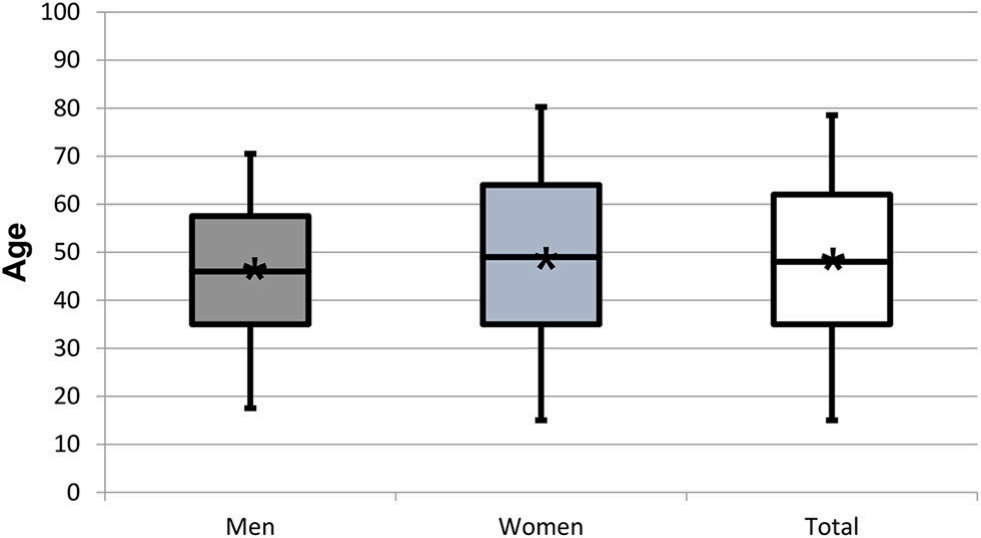
Box plots representing the age distribution by gender of the 682 patients who did not complete the WISE or did not consent to data use. Star symbol (^*^) indicates mean.

The population of the 952 patients completing the WISE (global sample) had a mean age of 44.8 ± 17.4 years (range: 10–90 years; 44.1 years). It consisted of 290 men (30.5%) with a mean age of 44.3 ± 16.5 years (range: 12–84 years; median 43 years) and 662 women (69.5%) of 45.0 ± 17.8 years (range: 10–90 years; median 46 years). The gender ratio therefore was 1:2.3. Mean ages between genders did not differ (*p* > 0.05) (Table 1; Figures 1, 2). The population of the 682 patients not completing the WISE or not giving consent had a mean age of 48.3 ± 18.3 years (range: 11–89 years; 48 years) and consisted of 213 men (31.2 %) with a mean age of 46.9 ± 17.4 years (range: 13–89 years; median 46 years) and 469 women of 48.9 ± 17.5 years (range: 11–89 years; median 49 years) (Figure 3). The gender ratio therefore was 1:2.2. Mean ages between the two populations did not differ (*p* > 0.05).

**Table 1:** Patient numbers (N) and distribution in the global sample stratified per decade and by gender.

	**10–19**		**20–29**		**30–39**		**40–49**		**50–59**		**60–69**		**70–79**		**80–89**		**90–99**		**Total**
	**M**	**W**	**M**	**W**	**M**	**W**	**M**	**W**	**M**	**W**	**M**	**W**	**M**	**W**	**M**	**W**	**M**	**W**	
*N*	20	49	36	104	64	110	60	124	53	123	35	87	19	60	3	4	0	1	952
Distribution (%)	2.1	5.1	3.8	10.9	6.7	11.6	6.3	13.0	5.6	12.9	3.7	9.1	2.0	6.3	0.3	0.4	0	0.1	100%

**Figure 4:**
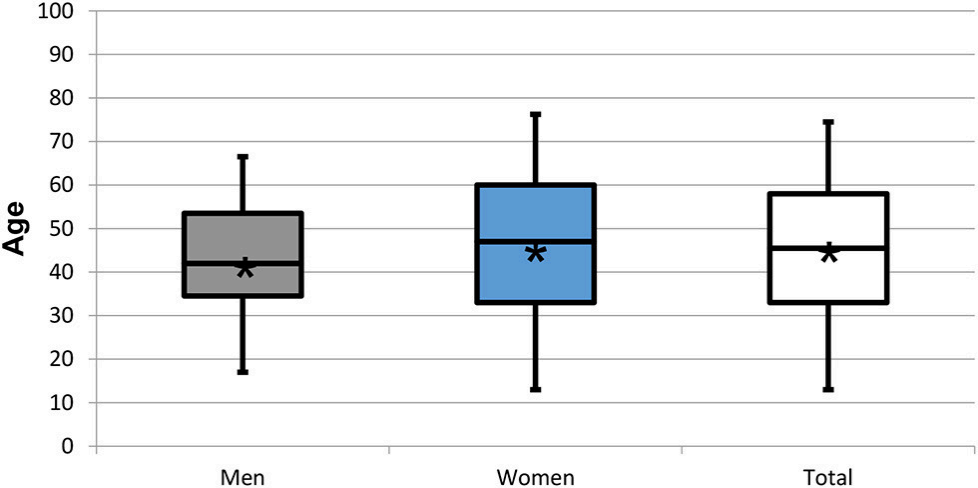
Box plots representing the age distribution by gender of the 352 patients responding positively to the screening question on insomnia and/or hypersomnia. Star symbol (^*^) indicates mean.

**Figure 5:**
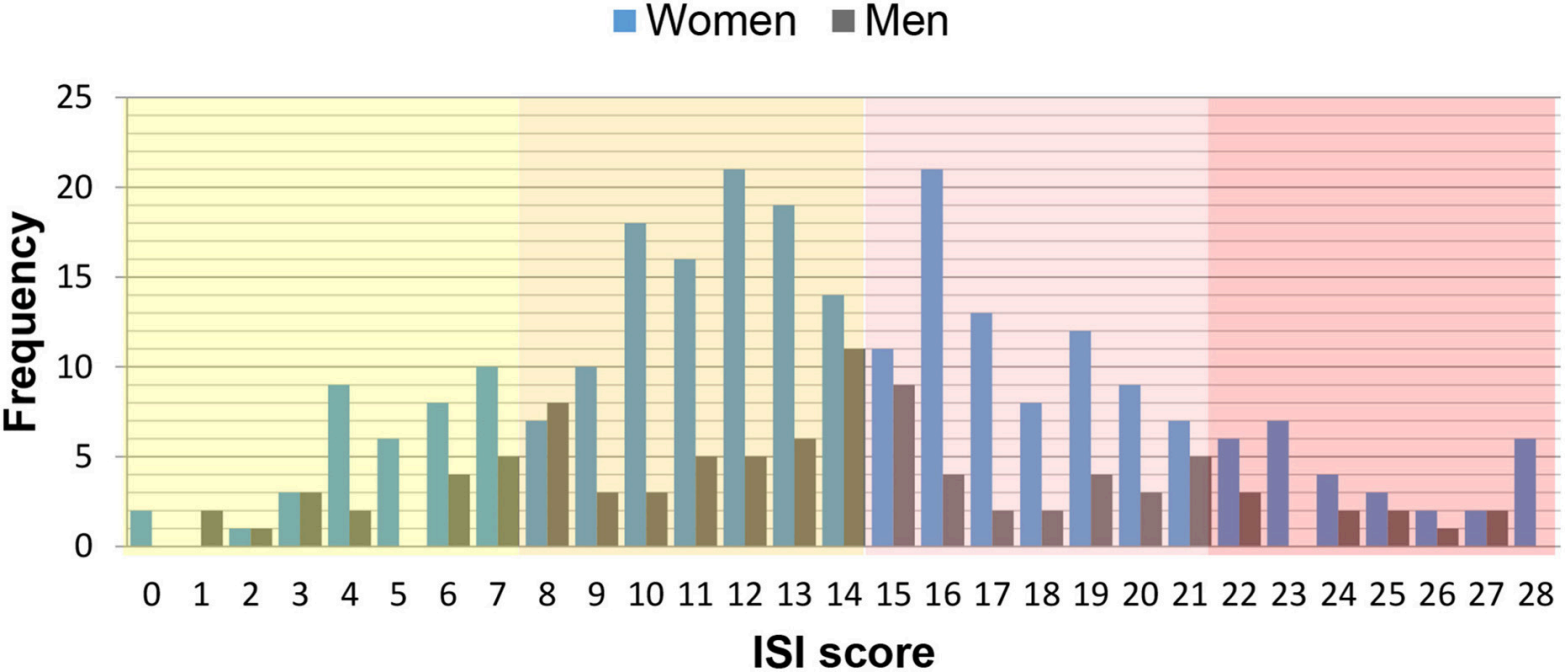
Distribution of insomnia severity index (ISI) scores stratified by gender. Background colors reflect ISI categories: not clinically significant (yellow), subthreshold (orange), moderate (pink), severe (red).

Three hundred and fifty-two (37.0%) patients with a mean age of 45.8 ± 16.7 years (range: 13–88 years; 45.5 years) reported “trouble falling or staying asleep or sleeping too much” on the WISE checklist and the ISI was presented to these. The 97 men (10.2%) in this group had a mean age of 43.5 ± 13.8 years (range: 17–80 years; median 42 years) and the 255 women (26.8% of global sample) of 46.7 ± 17.6 years (range: 13–88 years; median 47 years). The gender ratio therefore was 1:2.6. Neither age nor gender distribution differed between patients with insomnia and all patients (*p* > 0.05) (Table 2; Figures 2, 4). For the four ISI categories, the distribution of men and women, the gender ratio (in round brackets), and the proportions within each gender [in square brackets] were as follows: not clinically significant 15.9% (1:2.3) [1.1:1], subthreshold 41.5% (1:2.6) [1:1], moderate 31.2% (1:2.8) [1:1.1], and severe 11.4% (1:3) [1:1.2] (Table 2; Figure 5).

**Table 2:** Patient numbers (N), distribution, proportion within gender, and prevalence of insomnia stratified by ISI categories and by gender.

									**Clinically relevant**											
	**Not clinically significant**				**Subthreshold**				**Moderate**				**Severe**				**Total**			
	**M**	**W**	**T**	**M/W**	**M**	**W**	**T**	**M/W**	**M**	**W**	**T**	**M/W**	**M**	**W**	**T**	**M/W**	**M**	**W**	**T**	**M/W**
*N*	17	39	56		41	105	146		29	81	110		10	30	40		97	255	352	
Distribution (%)	4.8	11.1	15.9	1:2.3	11.6	29.8	41.5	1:2.6	8.2	23	31.2	1:2.8	2.8	8.6	11.4	1:3	27.5	72.5	100	1:2.6
Proportion (%) within gender	17.5	15.3		1.1:1	42.2	41.2		1:1	29.9	31.8		1:1.1	10.3	11.8		1:1.2			
Prevalence (%) (952 pts)	1.8	4.1	5.9	1:2.8	4.3	11.0	15.3	1:2.6	3.0	8.5	11.5	1:2.8	1.1	3.2	4.3	1:2.9	10.2	26.8	37	1:2.6
Prevalence (%) within gender (290 M, 662 W)	5.9	5.9		1:1	14.1	15.9		1:1.1	10	12.2		1:1.2	3.5	4.5		1:1.3			

*M/W, ratio men to women; pts, patients*.

**Figure 6:**
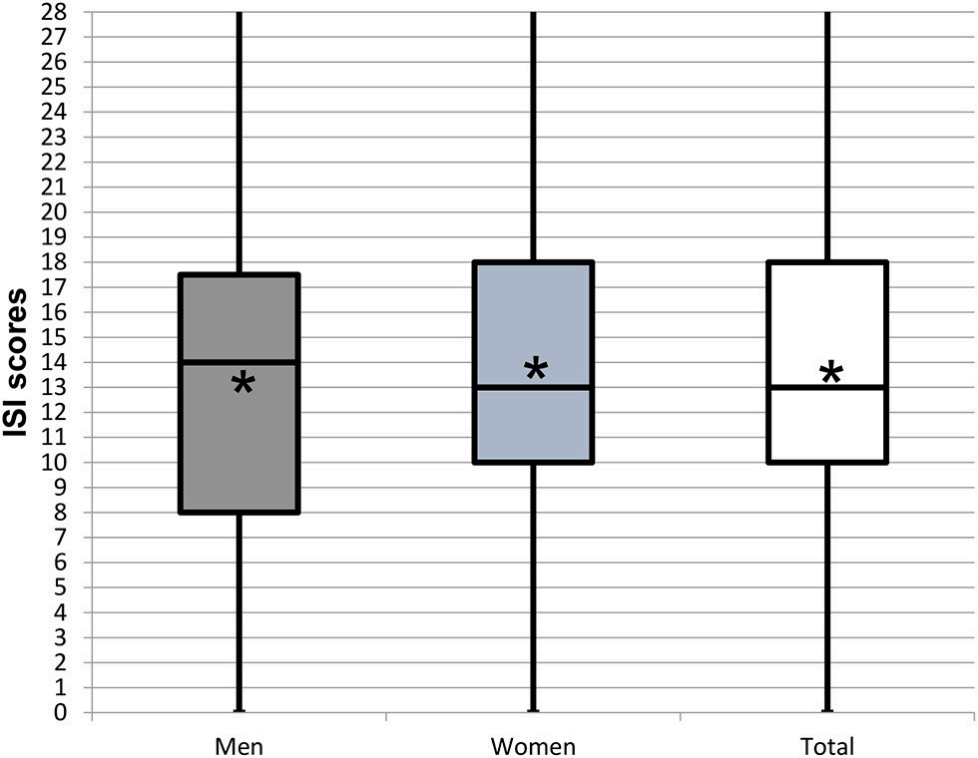
Box plots representing the distribution of the Insomnia Severity Index (ISI) scores reported by patients experiencing insomnia by gender. Star symbol (^*^) indicates mean.

The distribution of ISI scores, its gender ratios (in brackets), and its ratios within each gender [in square brackets] according to increasing severity grades were: 5.9% (1:2.8) [1:1], 15.3% (1:2.6) [1:1.1], 11.5% (1:2.8) [1:1.2] to 4.3% (1:2.9) [1:1.3] (Table 2). The mean ISI score was 13.8 ± 6.0 (men 13.4 ± 6.2; women 13.9 ± 6.2 (Figure 6).

**Figure 7:**
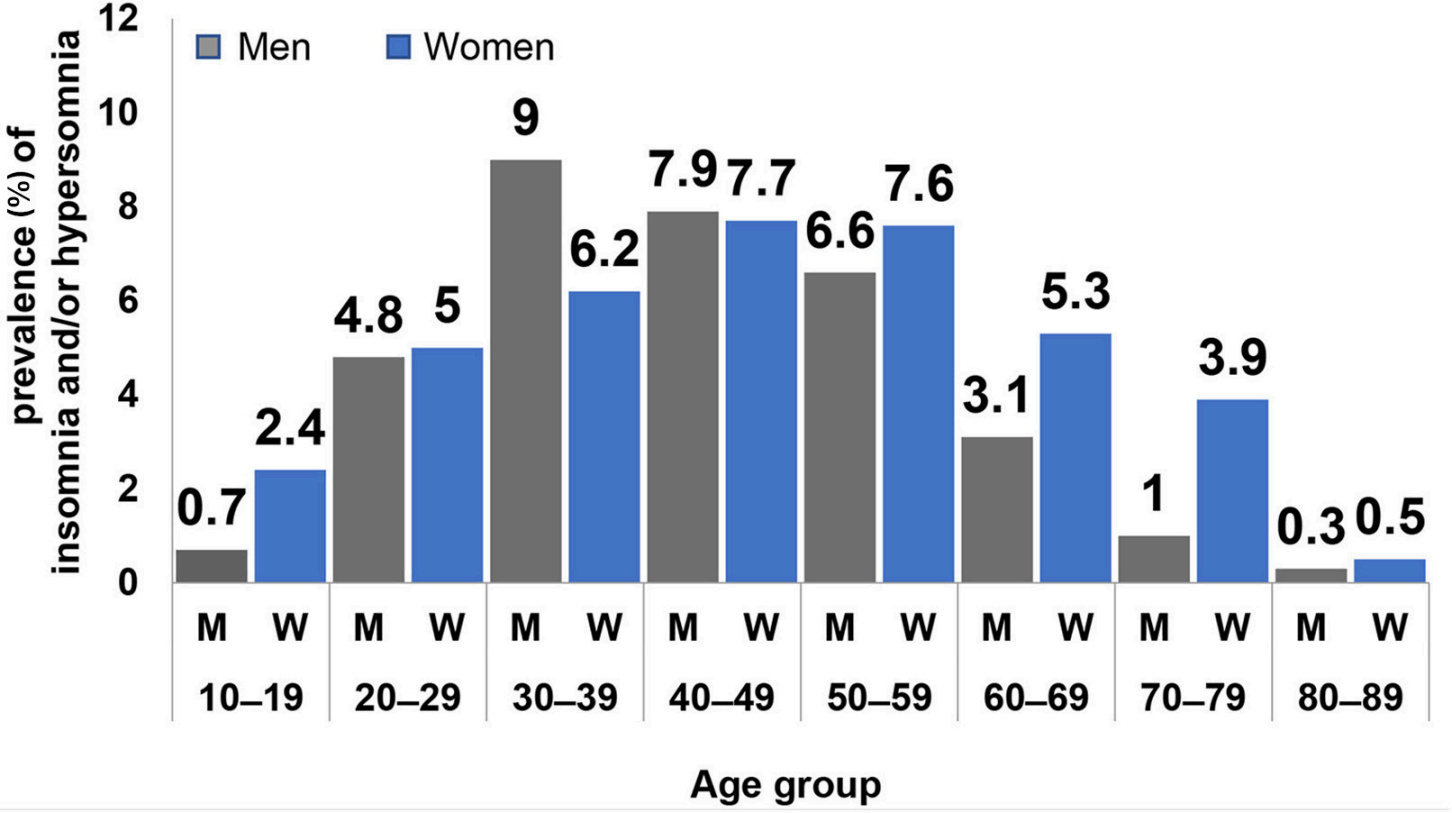
Prevalence (%) of insomnia and/or hypersomnia, stratified by men (M), and women (W) as well as by age group (decades).

The patients responding positively to the screening question on insomnia and/or hypersonia are listed in Table 3, sorted by distribution and proportion within gender per decade and by gender. This table also lists the prevalences according to all patients, to gender groups, and to gender of each decade in the global sample. The prevalence data in this table and Figure 7 reveal that 1.9% of teenagers and adolescents experienced insomnia. Among males, insomnia was most prevalent in the 4th and 5th decade (2.7 and 2.4%, respectively) while prevalence peaked in women in the 5th and 6th decades (5.4 and 5.3%, respectively). The ratio of the prevalence within gender and within gender per decade (in brackets) was: 2nd 1:3.4 (1:3.3), 3rd 1:1 (1.2:1), 4th 1.5:1 (1.1:1), 5th 1:1 (1:1.1), 6th 1:1.2 (1:1.1), 7th 1:1.7 (1:1.6), 8th 1:3.9 (1:2.7), and 9th 1:1.7 (1:2.6) (Table 3; Figure 7).

**Table 3:** Patient numbers (N), distribution, proportion within gender, and prevalence of patients responding positively to the screening question on insomnia and/or hypersomnia, stratified per decade and by gender.

	**10–19**			**20–29**			**30–39**			**40–49**			**50–59**			**60–69**			**70–79**			**80–89**		
	**M**	**W**	**T**	**M**	**W**	**T**	**M**	**W**	**T**	**M**	**W**	**T**	**M**	**W**	**T**	**M**	**W**	**T**	**M**	**W**	**T**	**M**	**W**	**T**
*N*	2	16	18	14	33	47	26	41	67	23	51	74	19	50	69	9	35	44	3	26	29	1	3	4
Distribution (%)	0.6	4.5	5.1	4.0	9.4	13.4	7.4	11.6	19.0	6.5	14.5	21.0	5.4	14.2	19.6	2.6	9.9	12.5	0.9	7.4	8.2	0.3	0.9	1.1
			**M/W**			**M/W**			**M/W**			**M/W**			**M/W**			**M/W**			**M/W**			**M/W**
Proportion (%) within gender	2.1	6.3	**1:3**	14.4	12.9	**1.1:1**	26.8	16.1	**1.7:1**	23.7	20.0	**1.2:1**	19.6	19.6	**1:1**	9.3	13.7	**1:1.5**	3.1	10.2	**1:3.3**	1.0	1.2	**1:1.2**
Global prevalence (%) (952 pts)	0.2	1.7	**1:8.5**	1.5	3.5	**1:2.3**	2.7	4.3	**1:1.6**	2.4	5.4	**1:2.3**	2.0	5.3	**1:2.7**	0.9	3.7	**1:4.1**	0.3	2.7	**1:9**	0.1	0.3	**1:3**
Prevalence (%) within gender (290 M, 662 W)	0.7	2.4	**1:3.4**	4.8	5.0	**1:1**	9.0	6.2	**1.5:1**	7.9	7.7	**1:1**	6.6	7.6	**1:1.2**	3.1	5.3	**1:1.7**	1.0	3.9	**1:3.9**	0.3	0.5	**1:1.7**
Prevalence (%) within gender per decade (Table 1)	10	32.7	**1:3.3**	38.9	31.7	**1.2:1**	40.6	37.3	**1.1:1**	38.3	41.1	**1:1.1**	35.9	40.7	**1:1.1**	25.7	40.2	**1:1.6**	15.8	43.3	**1:2.7**	33.3	75	**1:2.6**

*M/W, ratio men to women; pts, patients. Male to female ratios are highlighted in bold*.

**Figure 8:**
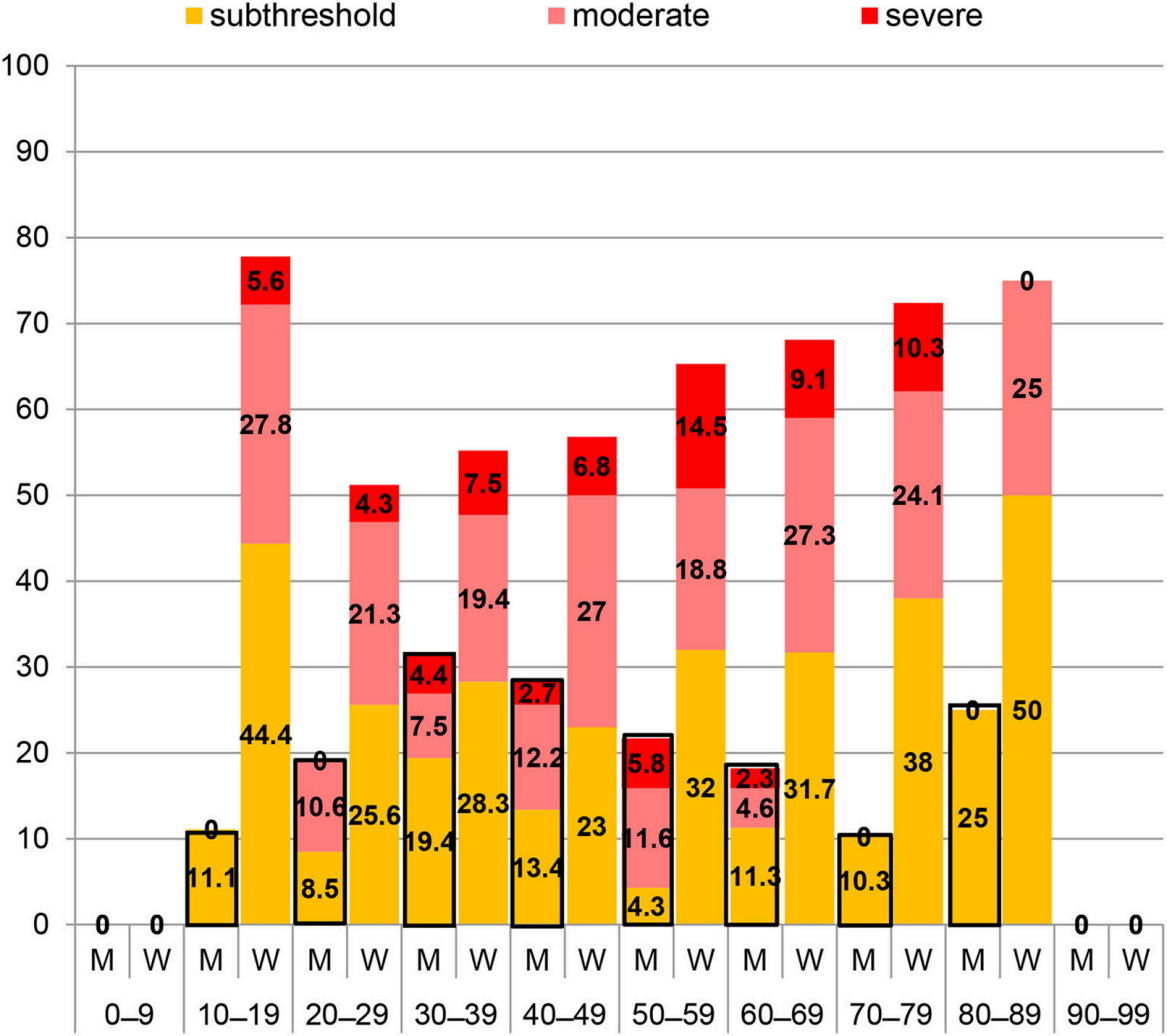
Distributions of Insomnia Severity Index (ISI) categories including only scores ≥ 8 per decade among 97 men (M) and 255 women (W).

Figure 8 shows the distributions of the three ISI categories (scores ≥ 8) per decade and by gender. The severe grade was present in women from the 2nd to 8th decade, ranging from 4.3% (3rd decade) to 14.5% (6th decade) and moderately severe from the 2nd to 9th decade, ranging from 18.8% (6th decade) to 27.8% (2nd decade). In men, severe insomnia was present from the 3rd to 7th decade, ranging from 2.3% (7th decade) to 4.4% (4th decade) and moderately severe from the 3rd to 7th decade, ranging from 4.6% (7th decade) to 12.2%(5th decade).

## Discussion

In this paper, we analyzed complaints of insomnia in a large sample of 952 patients seeking care at an orofacial pain unit, covering a broad age range. A first main study finding was that 37.0% of those patients reported sleep disturbance in the form of insomnia and/or excessive daytime sleepiness (hypersomnia). Secondly, women across a broader age range than men were affected by insomnia. Thirdly, when taking into consideration the proportions within gender, the ratios of insomnia between men and women were almost equal in young and middle-aged adults, yet higher in female teenagers and adolescents as well as older women.

For the three populations, namely the global sample completing the WISE, the sample that did not complete the WISE or did not consent to data use, and the patients reporting insomnia, the mean ages of 44.8 ± 17.4, 48.3 ± 18.3, and 45.8 ± 16.7 years, respectively, were not statistically different (*p* > 0.05) (Table 1; Figures 1, 2). Also the gender ratios of 1:2.3, 1:2.2, and 1:2.6, respectively, were similar among the three populations. This mean age and the gender ratio corresponds to a patient sample of our clinic previously analyzed with a different study question (42). The unequal gender distribution of the global sample is important and can be accounted for by calculating within gender ratios for men and women. Accordingly, focusing on the ratios of the proportions within each gender in Table 2, they were almost equal (1:1.1 for the lower three severities and 1:1.2 for the highest severity), meaning that the proportion of men and women in all ISI categories hardly differed. Yet, with increasing severity, the prevalence of insomnia slightly increased in women compared to men (from 1.1:1 to 1.1:3), although the mean scores did not significantly differ between gender (Figure 6). Among all patients reporting insomnia, moderate and severe grades were reported by almost half (42.6%), representing a combined prevalence of 15.8% of these two categories. This means that one in six patients suffered from clinically relevant insomnia (Table 2; Figure 5). If the threshold criterion of an ISI score of 8 were considered, this number would double to 1 in three patients (31.1%).

The proportions within each gender had also relatively balanced ratios in the population of the 3rd, 5th, 6th, and 9th decade (Table 3). Men experienced proportionately more insomnia only in the 4th decade (1.5:1), whereas a reverse pattern was seen in the 2nd, 7th, and 8th decade with values of 1:3.4, 1:1.7 and 1:3.9, respectively. This means that younger and older women experienced insomnia disproportionately more than men (Figure 7).

The distribution of the three ISI categories (scores ≥ 8) per decade and by gender is displayed in Figure 8. While ISI scores in men peaked in the 4th decade and gradually declined thereafter, a reverse pattern was observed in adult women with a trend toward increasing scores with older age. In other words, with increasing age, women tended to have only slightly more insomnia yet of higher severity. Notably, severe insomnia was not reported by women in the 9th decade. On the opposite, the largest proportion of scores ≥ 8 was reported by female teenagers and adolescents, yet mainly due to the relatively high percentage of subthreshold insomnia.

Comparing our results to other studies is difficult due to the restricted use of the ISI or other validated tools for analyzing insomnia in large cohorts. We mostly identified studies using other types of self-reports that were not validated for grading insomnia severity. Hence, insomnia prevalence's ranging from 5 to 50% reported in those works have to be interpreted with caution (18–25). An exception was the report by Jank et al. who employed the ISI for analyzing insomnia in a mixed group with and without pain complaints (40). In this sample of 570 patients from Austria, insomnia prevalence was 29.1%, with 55 subjects (9.6%) complaining of clinically relevant insomnia. The mean age of this population was slightly higher (50.8 ± 18.7 years). Gender ratios were not calculated. Another study investigated insomnia in 481 Korean patients complain of low back pain (43). The mean age of the study sample was 58.2 ± 16.7 (range 20–90). The gender ratio was 1:1.5 compared to 1:2.3 in our sample. The mean ISI score was 7.5 ±7.1 and thus much lower compared to 13.8 ± 6 observed here. The distribution of insomnia among the four ISI categories compared to ours (in brackets) in ascending severity was: 57.0 (5.9), 23.0 (15.3), 14.7 (11.5), and 5.3 (4.3). Thus, the proportion of clinically relevant insomnia was similar. However, no gender ratios were provided that would allow an estimation of the gender related distribution. In 6,205 Swedish individuals older than 65 years with no, subacute and chronic pain, the proportion of moderate and sever insomnia was 35.1 and 4.3%, respectively (41). In this cohort, a mean ISI score of 10 was observed in the group experiencing subacute pain (*N* = 510) and 10.9 in the chronic pain group (*N* = 2,790), both of which are lower than the mean ISI score of 13.8 identified in our sample of OPD. None of the above-mentioned studies analyzed insomnia severity across the life span.

Our analysis has some noteworthy limitations: it is cross-sectional, i.e., insomnia was captured at one point in time. Hence, the results do not allow inferring potential time-related dynamics. As only few studies report high quality prevalence data of insomnia in persons suffering from pain (40, 41), the inclusion of age and gender paired controls would be an interesting aspect to be considered in future studies. As we aimed at reporting insomnia in a representative sample of our clinic population, we also included persons aged 10–20 years, even though the ISI is not specifically validated for this age group.

Also, no statements can be made regarding possible associations of insomnia complaints with the patients' chief complaints and/or diagnoses, co-morbidities (e.g., psychopathologies) or other confounding factors (e.g., snoring), since we did not take these aspects into account. Specifically, the assessment of somatic and/or psychological co-morbidities known to influence sleep (e.g., tinnitus, wide-spread pain, anxiety, depression, distress, catastrophic rumination, neuroticism, obsessive-compulsive, or post-traumatic stress disorder) would have offered information on possible cause-effect relationships, yet this effort was outside the scope of this work. Finally, insomnia could also be secondary to the use of medication or substance use, both of which were not controlled as a co-factor in the study.

Nonetheless, future research should focus on cross-sectional and longitudinal interactions of insomnia, psychopathology and other associated factors in patients with OPD (44).

## Conclusions

This is the first study reporting on insomnia in a large sample of patients referred to an orofacial pain unit. 37.0% of patients responded positively to a screening question for insomnia and/or hypersomnia. For almost half of them, insomnia was moderate to severe. The gender ratio was almost equal throughout adulthood, yet younger and older women were more affected and experienced higher severity than men.

## Ethics Statement

According to Swiss law, the analysis of strictly anonymized data does not require approval by an ethics committee.

## Author Contributions

MM and DE: study design, data analysis, manuscript writing. NL: data collection. BS: study design, data collection. AW: data colectivo. AG: study design.

### Conflict of Interest Statement

The authors declare that the research was conducted in the absence of any commercial or financial relationships that could be construed as a potential conflict of interest.
